# High prevalence of neurocognitive disorders observed among adult people living with HIV/AIDS in Southern Ethiopia: A cross-sectional study

**DOI:** 10.1371/journal.pone.0204636

**Published:** 2019-03-18

**Authors:** Megbaru Debalkie Animut, Muluken Bekele Sorrie, Yinager Workineh Birhanu, Manaye Yihune Teshale

**Affiliations:** 1 Arbaminch University College of Medicine and Health Sciences Department of Nursing, Arbaminch, Ethiopia; 2 Arbaminch University College of Medicine and Health Sciences Department of Public Health, Arbaminch, Ethiopia; 3 Bahir Dar University Colleges of Medicine and Health Sciences School of Nursing, Bahir Dar Ethiopia; 4 Arbaminch University College of Medicine and Health Sciences Department of Public Health, Arbaminch, Ethiopia; The University of New South Wales, Neuroscience Research Australia, AUSTRALIA

## Abstract

**Background:**

Comprehensive care given to people living with HIV/AIDS is improving over time; however, their concurrent cognitive illness is still ignored, under screened and treated particularly in developing countries. And this problem is also striking in Ethiopia. Therefore, the objective of this study was to assess HIV-associated neurocognitive disorders and associated factors among adult people living with HIV/AIDS.

**Methods:**

An institution based cross sectional study was conducted from April to May, 2017 at Gamo Gofa zone public Hospitals. International HIV Dementia Scale was used to screen HIV associated neurocognitive disorders. Logistic regression analysis was used to assess predictors of neurocognitive disorders.

**Result:**

A total of 684 study participants were included in this study with a response rate of 98%. Among them, 56% were females while 44% were males. The mean (±SD) age of the participants was 38.8±8.8years. The screening prevalence of HIV-associated neurocognitive disorder was 67.1% (95% CI; 63.6, 70.5). Body mass index 16 kg/m^2^ (AOR 4.389 (1.603–12.016)), being married (AOR 0.377 (0.213–0.666), unemployment status (AOR 3.181 (1.752–5.777) and being in WHO clinical stage T3 category/advancing stages of the disease (AOR 3.558 (1.406–9.006) were the key predictors of HIV-associated neurocognitive disorders among people living with HIV/AIDS.

**Conclusion:**

In this study the screening prevalence of HIV-associated neurocognitive disorder is higher than the earlier reports in Ethiopia and Africa. This indicates that early screening strategies and policies for cognitive health in people living with HIV/AIDS should be given a top priority.

## Background

One of the most devastating health problems facing the human race in the 21 century is the Acquired Immunodeficiency Syndrome (AIDS) caused by the Human Immunodeficiency Virus (HIV) [1]. Sub-Saharan African (SSA) countries are highly affected by this disease, accounting 80% of the cases, despite the continent constituting only 11–12% of the world’s population [1, 2]. HIV is a neurotropic virus that affects the sub-cortical structure of the brain [3]. Cognitive impairment of patients due to HIV is called HIV-associated neurocognitive disorder (HAND) [4, 5]. It results in a defect in speed of problem solving ability, loss of memory and attention [6]. HAND is a collective term which comprises 3 levels of illness that ranges from asymptomatic neurocognitive impairment to minor neurocognitive disorder and frank dementia [1, 7–10]. Even though severe form of HAND like dementia is decreasing after the introduction of combined highly active antiretroviral therapy (cART), mild and asymptomatic form of neurocognitive disorder is still prevalent and a major problem globally [4, 10–17]. Despite cART reduces the prevalence of HAND, accessing anti-retroviral therapy (ART) in sub Saharan countries is a big problem so far. Moreover the late presentation of patients in the follow-up clinic makes them vulnerable to HAND and other HIV related complications [18–20].

The prevalence of HIV associated neurocognitive disorder (HAND) in adult people living with HIV/AIDS (PLWHA) ranges from 19% to 52% in developed [19, 20], and 14% to 64% in developing countries [9, 10].

HAND predisposes HIV infected patients for poor adherence, unsafe sex, substance abuse, alcohol addiction, lost to follow up and worsens their quality of life. All these factors can accelerate HIV symptoms, their progression towards AIDS defining opportunistic infections and death [7, 9, 21].

Studies carried out in Singapore, Nigeria, Cameroon, Botswana, Malawi and Dessie Ethiopia indicated that being female, old age and low educational status were an independent risk factor for HIV associated neurocognitive disorder [2, 6, 9, 10, 18, 21–23], whereas, studies carried out in Brazil, Singapore and Northern Nigeria indicated that CD4 count less than 500 cells/mm3 was associated with HIV-associated neurocognitive disorder [2, 6, 18, 24]. Moreover, a study done in South Africa in 2013 showed that highly active anti-retroviral treatment (HAART) naïve and late clinical stage of the illness was factors affecting HIV-associated neurocognitive disorder [20, 23]. Body mass index, depression and alcohol abuse were also found to be associated with HIV-associated neurocognitive disorder in Uganda [1]. In addition to the above factors, the presence of opportunistic infections and poor medication adherence were associated with HIV-associated neurocognitive disorder [8, 25].

In Ethiopia, particularly in the study area, there is deficient information regarding the prevalence of HIV-associated neurocognitive disorder and its associated factors among peoples living with HIV/AIDS though there are many people living with HIV/AIDS[8].

Therefore, this study will help to determine the prevalence and the most important associated factors which have an impact on HIV-associated neurocognitive disorders among peoples living with HIV/AIDS.

## Methods

### Study design, setting and area

An institution based cross-sectional study was conducted from April to May 2017 in the Gamo Gofa zone, which is located at a distance of 505 km from Addis Ababa (the capital of Ethiopia). It is administratively organized in 15 Woreda, 2 administrative towns, 34 urban and 452 rural kebeles. The total populations are 2,040,972 according to the 2017 Zonal report. There are 3 public hospitals, 73 health centers and 471 health posts in the Gamo Gofa zone currently functional according to unpublished zonal health officials’ report 2017.

### Study population

The target population was PLWHA aged between 18 and 64 years who had treatment follow up at Gamo Gofa zone public Hospital ART clinics. Those selected PLWHA who was getting ART service during the data collection period were included in the study. PLWHA who were seriously ill during the study period, known psychiatric and neurological problems (depression, psychosis and epilepsy/seizure disorders) were excluded from the study.

### Sample size and sampling procedures

Sample size was determined by taking the two significant variables from previous studies which was CD4 count less than 500 cells/mm^3^ and having a primary educational level or less in a study done at South Wollo, Ethiopia [8, 22] with 80% power, 0.36 odds ratio, 95% CI and a none response rate of 10%. Based on this assumption, the final sample size was 697. Systematic random sampling was employed to select study participants. From 3 Hospitals, samples were allocated to each Hospital proportionally based on their number of PLWHA.

Finally, PLWHA in every 3^rd^ PLWHA from their regular follow up in the hospital was enrolled in the study by systematic random sampling method.

### Data collection tools and procedures

A structured questionnaire was used to collect data on socio- demographic characteristics of the participants and clinical factors of HIV/AIDS (WHO stages, CD4 count…etc.). The WHO staging system uses the laboratory method to categorize the immune status of HIV/AIDS patients by their total lymphocyte counts (CD4) and clinical markers believed to have prognostic significance resulting in four categories. WHO stage I, II, III & IV before the initiation of cART and WHO stage T1, T2, T3 & T4 six months after cART initiation. T stands for treatment. This staging system has proven reliable for predicting morbidity and mortality in HIV/AIDS patients especially in developing countries where sophisticated laboratory investigations are inaccessible [26]. Other variables that were important for the objective were extracted from the patient's chart.

International HIV Dementia Scale (IHDS) was used as a screening tool to identify individuals who are at risk of HAND. The presence of HAND was screened by an IHDS score of <9.5 in this study. This tool has been validated in Sub-Saharan African countries, particularly in South Africa, Cameroon, Botswana, Uganda and Ethiopia and found to have good psychometric properties in African populations with sensitivity of 88% and 80% and specificity of 50% and 55% at a cutoff 10 or less [1, 9, 10, 20]. HAND consists of the three subsets of tests, namely motor speed, psychomotor speed and memory-recall test. The procedure was as follows:

#### Motor Speed test

It was measured by instructing the participant to open and close the first two fingers of his/her non-dominant hand as widely and as quickly as possible over a 5-second period. The score was given as follows (4 = 15 in 5 seconds 3 = 11–14 in 5 seconds, 2 = 7–10 in 5 seconds, 1 = 3–6 in 5 seconds, 0 = 0–2 in 5 seconds).

#### Psychomotor speed test

In this procedure participant was ordered to perform the following activities by their non-dominant hand within 10 seconds as fast as possible. First clench their fist on a flat table secondly put the hand flat on the table with the palm down and finally make their flat hand perpendicular to the table on the fifth digit side. This procedure was demonstrated to the participant by the data collector and the participant was allowed to practice the procedure correctly before actual 10 second sequence was performed. The number of sequences correctly performed within 10 seconds up to a maximum number of 4 is scored (4 = 4 sequences in 10 seconds, 3 = 3 sequences in 10 seconds, 2 = 2 sequences in 10 seconds, 1 = 1 sequence in 10 seconds and 0 = unable to perform).

#### Memory-Recall

In this procedure participants were asked to repeat four words after reciting by the data collector. The words are repeated by the data collector until the participant can repeat all four words correctly. The participants were asked to recall the four words after the above two procedures. For words not recalled, the participants were reminded with a ‘semantic’ clue as follows: Animal (Cow), a piece of cloth (T-shirt), Vegetable (cabbage), and color (Blue). 1 point was given for each word spontaneously recalled and 0.5 points for each correct answer after semantic clues and the maximum point were scored out of 4. Finally IHDS score was calculated by adding all three subsets out of 12 points and participants who scored <9.5 were screened as having a risk of HAND in this study.

#### Adherence of patients to their medication was assessed using Morsiky-8 item scale (MMAS)

The MMAS-8 is a self-report questionnaire with 8 questions (items). Items 1 through 7 have response choices “yes” or “no” whereas item 8 has a 5-point Likert response choices. Each ‘‘no” response is rated as ‘‘1” and each ‘‘yes” is rated as ‘‘0” except for item 5, in which each response ‘‘yes” is rated as ‘‘1” and each ‘‘no” is rated as ‘‘0”. For item 8, if a patient chooses a response ‘‘0”, the score is ‘‘1” and if they choose response ‘‘4”, the score is ‘‘0”. Responses ‘‘1, 2, 3” are respectively rated as ‘‘0.25, 0.75, 0.75”. Total MMAS-8 scores can range from 0 to 8 and have been categorized into three levels of adherence: high adherence (score = 8), medium adherence (score of 6 to < 8), and low adherence (score< 6) [8, 27].

#### Alcohol use disorder identification test

The Alcohol Use Disorder Identification Test (AUDIT) is a 10-item instrument designed by the World Health Organization to screen excessive consumption, hazardous, and harmful use of alcohol. The instrument has been validated in many countries, including Sub-Saharan African countries. A score of 8 and above is indicative of either harmful use or dependence [2]. And a cut of point > = 8 was used in this study.

#### Drug abuse screening test

Drug Abuse Screening Test (DAST) instrument is designed to screen for drug use other than alcohol [28].The DAST-10 version was used in this study and is answered in a ‘‘yes or no” pattern. A score of 3 and above is indicative of a problem with psychoactive substance abuse [2].

### Data quality management

Data collectors were trained for two days by doing standardization exercise in order to minimize errors. The questionnaire was developed in English and then translated into Amharic (local language) and back to English then review was made for its consistency. Data was collected after pretest has been conducted on 35 (5%) of PLWHA from non-selected health institutions. The principal investigator made day to day on-site supervision during the whole period of data collection. The collected data were reviewed and checked for completeness, accuracy and consistency by the investigator.

### Data management and analysis

Data entry and cleaning was made using Epidata 3.1 and exported to SPSS software package version 20 for analysis.

Cross-checking and data cleaning was carried out by running frequencies of each variable. For specific objective one, descriptive statistical methods such as frequencies, percentages, proportion with 95% C.I has been used. Mean and standard deviation was also used to summarize various characteristics of the participants. For specific objective two, cross tabulation and bivariable logistic regression were used to explore the relation between the outcome variable and the different independent variables using crude odds ratio with 95% C.I. Finally, to determine the independent factors associated with the outcome variable, multivariable logistic regression model were done, and presented using adjusted odds ratio (AOR) with 95% confidence interval. Variables with P-value < 0.2 of the bivariable analysis were taken in to the multivariable logistic regression model. Model fitting was checked using log likelihood and Hosmer-Lemeshow test. Finally, variables with P < 0.05 in the multivariable analysis were considered as significant.

## Results

### Socio-demographic characteristics

Six hundred eighty four (684) people living with HIV/AIDS were involved in this study, making the response rate of 98%. The mean age of the respondents was 38.8±8.8 years. Among the study participants majority 383 (56.0%) were females and 284 (41.5%) were widowed (Table 1).

**Table 1:** Distribution of PLWHA by their socio-demographic characteristics at Arbaminch general hospital, Chencha & Saula district hospitals ART clinic, 2018.

Variables		Frequency (n = 684)	Percent
Sex	Male	301	44.0
	Female	383	56.0
Age	18–25	27	3.9
	26–35	224	32.7
	36–45	255	37.3
	46–56	164	24.0
	>56	14	2.0
Marital status	Single	143	20.9
	Married	142	20.8
	Widowed	284	41.5
	Divorced	115	16.8
Residence	Urban	594	86.8
	Rural	90	13.2
Religion	Orthodox	344	50.3
	Protestant	276	40.4
	Muslim	58	8.5
	Others	6	0.9
Educational status	Unable to read &write	130	19.0
	Able to read &write	206	30.1
	Primary	216	31.6
	Secondary & above	132	19.3
Occupation	Employed	624	91.2
	unemployed	60	8.8
Monthly income(ETB)	≤585	166	24.3
	586–1650	263	38.5
	1651–3145	181	26.5
	≥3146	74	10.8

Key: ETB = Ethiopian Birr (Ethiopian currency).

### Clinical status

Most of the participants 589 (86.1%) were in WHO clinical stage T1 category and their mean CD4 count was 610 ± 278 cells/mm^3^. And 95.6% of the participant had no any history of central nervous system related opportunistic infections. Adherence of participants with their ART medication was assessed using Morsiky-8 item scale (MMAS). And MMAS ranges from 0–8 points. The majority of the participants 408 (59.6%) were low adherent to their ART medication i.e. they scored <6 points by this scale (Table 2).

**Table 2:** Distribution of PLWHA by their clinical status at Arbaminch general hospital, Chencha & Saula district hospitals ART clinic, 2018.

Variables		Frequency(n = 684)	Percent
BMI(Kg/m^2^)	18.5–24.9	486	71.1
	17.5–18.5	74	10.8
	16–17.5	104	15.2
	<16	20	2.9
WHO clinical stage	T1	589	86.1
	T 2	33	4.8
	T 3	26	3.8
	T 4	36	5.3
Recent CD4 count(cells/mm^3^)	<500	217	31.7
	> = 500	467	68.3
Duration with ARV treatment (Yrs.)	≤5	360	52.6
	>5	324	47.4
History of OIS	CNS toxoplasmosis	26	3.8
	Cryptococal meningitis	4	0.6
	No OI	654	95.6
Presence of comorbid illnesses	DM	41	6.0
	Hypertension	92	13.5
	Cardiac problem	6	0.9
	No	545	79.7
Adherence to their ART	High adherent	171	25.0
	Medium adherent	105	15.4
	Low adherent	408	59.6

Key: ART = antiretroviral treatment, ARV = antiretroviral BMI = body mass index, Kg/m^2^ = kilogram per meter square, DM = diabetic mellitus, OI = opportunistic infections, Yrs = years

### Alcohol and other drug usage pattern

Alcohol use disorder identification test was used to screen excessive consumption, hazardous, and harmful use of alcohol. And in this study majority 624 (91.2%) of the participants never used alcohol excessively, hazardously or harmfully. Drug abused screening test (DAST-10) was also used to screen participants for their drug usage other than alcohol and most of the participants 628 (91.8%) scored between 0–3 which is non-drug abuse.

### Prevalence of HIV associated neurocognitive disorder (HAND)

Among the study participants who have been screened for HAND, 459 (67.1%, 95% CI; (63.6, 70.5)) tested positive for HAND. Procedural implementation of international HIV dementia scale (IHDS) was as follows: The first measurement on IHDS was timed finger tapping, on this part motor speed was assessed, 65 (9.5%) were scored 4 out of 4 which is normal. On the second part psychomotor speed measurement was assessed, of whom 109 (15.9%) had performed the sequential procedure, scoring 4 out of 4. Finally, memory recall was assessed and 174 (25.4%) had recalled all the four items without any clue scoring 4 out of 4.

### Factors associated with HAND

Bivariable and multivariable analysis showed that the association between HIV-associated neurocognitive disorder and marital status, body mass index, WHO clinical status and occupational status were significant. After controlling /adjusting other variables like (sex, alcohol abuse, religion, educational status, CD4 count, drug abuse, duration with ART, medication adherence, monthly income, comorbid illness, opportunistic infection history). The odds of HAND among married participant decrease by 62.3% compared to those with unmarried/single participants (AOR = 0.377 CI (0.213–0.666)**).**

In this study the likelihood of having the risk of HAND among peoples living with HIV/AIDS (PLWHA) was four times in those participants who had a body mass index of less than 16 kg/m^2^ than those participants who had a body mass index of 18.5–24.9 kg/m^2^ (AOR 4.389 CI (1.603–12.016).The odds of having HAND among unemployed participants were three times higher than those who were employed (AOR = 3.181 CI (1.752–5.777)). Similarly, late stage of the disease or being in WHO clinical staging T3 category was approximately four times more likely to have the risk of HAND (AOR = 3.558 CI (1.406–9.006)) when compared to those with WHO clinical staging T1 category (Table 3 below).

**Table 3:** Bivariable and multivariable analysis showing the association between socio-demographic and clinical related variables with HAND among PLWHA in Arbaminch general hospital, Chencha & Saula district hospital ART clinics, 2018.

Variables		HAND		COR	AOR

		YES	NO		
Marital status	Single	48	95	1	1
	Married	28	114	0.246(0.162–0.371)	0.377(0.213–0.666)*
	Widowed	104	180	0.578(0.454–0.736)	0.969(0.619–1.516)
	Divorced	45	70	0.643(0.442–0.935)	0.991(0.582–1.687)
Body mass index	18.5–24.9	162	324	1	1
	17.5–18.5	17	57	0.298(0.174–0.513)	0.589 (0.324–1.071)
	16–17.5	33	71	0.465(0.308–0.702)	0.907(0.560–1.467)
	<16	13	7	1.857(0.741–4.655)	4.389(1.603–12.016)*
Occupational status	Unemployed	37	23	0.673(0.619–0.731)	3.181(1.752–5.777)*
	Employed	188	436	1	1
WHO clinical stage	T1	186	403	1	1
	T2	11	22	0.500(0.242–1.031)	1.219 (0. 541–2.747)
	T3	18	8	2.250(0.978–5.175)	3.558(1.406–9.006)*
	T4	10	26	0.385(0.185–0.798)	0.745 (0.332–1.672)

Key

* = p < 0.05, Number of total participants n = 684.

## Discussions

Determination of the prevalence and associated factors of HIV-associated neurocognitive disorders among peoples living with HIV/AIDS by institutional based cross sectional study in southern Ethiopia public hospitals was the main focus of this study. At the base of this, the prevalence of HAND among people living with HIV/AIDS was 67.1%. And IHDS demonstration was highly associated with marital status, body mass index, occupational status and WHO clinical stage category.

The finding of this study was in line with the study done in Sub Saharan African population which showed the prevalence of HAND (64.4%) [1], and in Kenya (65.6%) [29].

However, the prevalence of HAND in the current study was higher than a cross-sectional study done in Brazil (53%) [23], in Botswana (38%) [10] Indonesia (51%) [30], Nigeria 30% [12], South Africa (25%) [31], South Asia (22.7%) [18] and in Ethiopia at Debre Markos North West (24.8%) [25], Dessie (36.4%) [8] and the Mekele referral hospital Northern Ethiopia 33.3% [32]. The differences may be accounted for the neurovirulence strain differences and hidden sociodemographic differences. In addition, most of the above studies used smaller sample size than this study. Above all the difference in the care of patients with HIV in different settings might be different and will contribute to the difference in prevalence.

On the other hand, the prevalence of HAND in this study was lower than a cross-sectional study conducted among 400 PLWHA in Bamenda, Cameroon (85%), at Bamenda Regional Hospital AIDS-treatment Centre [9].

The discrepancy in the prevalence rate could be explained by the existence of different neurovirulent clades in different countries. Clade A and D are the most common clades/sub types in sub Saharan African countries. But in Ethiopia clade C is the prevalent sub type [33]. Even though Clade C is a neurotropic virus like A and D, it is the least common cause of neurocognitive disorder than A&D [34]. The differences in the predictors for the development of HAND might also have an effect for the discrepancy since in the previous study the common predictor was stated as having primary education or less and having HIV symptoms but not in the current study. And the discrepancy can also be explained by sample size differences; in previous study lesser sample size (400), much less than the current study was used. In addition, other possible reasons for the differences between the present and other studies were mainly due to differences in the method used, differences in study populations, using different cutoff points in different studies, some inappreciable cultural and educational difference, and environmental factors. Furthermore, discrepancies may also be explained by the application of the IHDS which might need well-educated participant.

This study found out that, marital status was significantly associated with the development of HAND. Married participants were less likely to develop HAND than single individuals. This finding is also supported by a cross sectional study conducted among 225 participants in Tanzania which indicated that living alone than married is an independent risk factor for HAND [35]. The possible reason is that married couples can promote a healthy life like to motivate each other to exercise, eat healthfully, maintain social ties, smoke and drink less [36–39]. Furthermore, married couples can prevent modifiable risk factors for HAND [40, 41]. All these things could prevent the development of HAND. In single, widowed or divorced participants’ stress level will be high which further can affect nerve signaling in the brain and impair cognitive abilities [42]. In addition, people who are married tend to be financially better and stable than others, a factor that is closely associated with many aspects of human health [43].

In this study, body mass index of <16 kg/m^2^ was significantly associated with HAND which was nearly in line with USA Baltimore/Los Angeles sites of the Multicenter AIDS Cohort Study [44]. Severe adult malnutrition increases the incidence and severity of CNS opportunistic infections in PLWHA and in the long run patients will develop HAND [45]. Perhaps not surprisingly, the risk of different kinds of neurocognitive disorder/dementia can be increased by vitamin deficiencies, specifically B-12 [46], and other forms of Vitamin B which seems to be the vitamin most likely to potentiate the development of dementia if not consumed in adequate quantities [47, 48]. Malnutrition in PLWHA causes severe immune suppression and leads them to develop central nervous system opportunistic infections in cortical or subcortical structure of the brain, which facilitates neurocognitive disorder or dementia [45, 49]. This evidence is also supported by Cache County Dementia Progression Study which found out that nutritional status was associated with faster decline in cognitive dysfunction [50]. A longitudinal study done among 125 HIV/AIDS infected participants at The Miriam Hospital/Brown University, USA stated that malnutrition is a risk factor for neurocognitive disorder [51].

In the current study occupational status had significant association with HAND. Unemployment was an independent risk factor for the development of HAND. This study is supported by a cross sectional study done among 361 participants in Asia [52], a cohort study done in Thai [53] and cross sectional study among 604 participants in Ireland and Irish ART Clinic [54]. Employment makes a person to acquire new skills, participate in different organization and engage socially, all of these can give meaning to a person’s life and provide income [55]. In addition, these activities can boost a person’s cognitive health. Unfortunately, if they get unemployed, they will lose or limit at least employment driven cognitive health benefits [56, 57]. For people with HIV/AIDS money is used to buy different prophylactic drugs, vitamins, antiretroviral drugs and most importantly for food. In general, low socioeconomic status was independently associated with neurocognitive disorders in patients with HIV/AIDS [56, 58]. In the present study, there was statistically significant association between WHO clinical staging/late stage of the disease and HAND. This finding is supported by an observational cohort study done among 268 patients in the USA, demonstrate that this factor, was the predictive variables in the development of HAND [59]. An institution based cross sectional study conducted among 234 participants Ayder Comprehensive Specialized Hospital in Ethiopia, found out that late stage of the disease is an independent risk factor for neurocognitive disorder [32]. During late stage of the disease, there will be intense viremia both in the blood and Cerebro-spinal fluid (CSF). This can predispose patients for different central nervous system (CNS) related opportunistic infection. In addition the virus itself can replicate and multiply in different parts of the brain. Both mechanisms play a synergistic effect for the development of HIV associated neurocognitive disorders [60].

In our study adherence status of participants to their ART medication is low, even though we didn’t measure the serum concentration level of the drugs. But it wasn’t statically significant for HAND in this study. However, low adherence to ART medication is an independent risk factor for HAND in cross sectional studies done among 595 participants in South Wollo Ethiopia [8] and among 418 participants in Northern Nigeria [12]. This variable is also an independent risk factor for HAND in different studies conducted previously [11, 17, 61, 62].

But the association of HAND and adherence level has a reciprocal relationship in one systemic review [63]. A longitudinal study done among 225 participants in Boston, USA showed that adherence is highly affected by HAND [64]. Other similar studies also showed that HAND is a risk factor for low medication adherence [65–67].

Even though drug abuse wasn’t statically significant in this study, from 56 abuser participants 27 participants develop HAND. But this variable was an independent risk factor for HAND in a cross sectional study done among 423 participants in Deberemarkos Ethiopia [25]. It was also an independent risk factor for development of HAND in different Studies [68–70].

One of the limitations of this paper is that antiretroviral treatment regimen wasn’t recorded. It would have been better if we included this variable. Because, not all the drugs used to treat HIV penetrate the blood brain barrier (BBB) equally [7]. Regimens which contain drugs with better blood brain barrier crossing ability can suppress CNS HIV replication and prevents neurocognitive dysfunction [15, 71]. But in Ethiopia TDF-3TC-EFV are the most commonly used regimens.

Generally; the results of this study provide additional information on HAND particularly in the local areas where this study is done. The result of this study can also give some information on specific variables which affect HAND. Above all, the finding of this study will contribute about the burden and risk factor of HAND to the scientific world particularly in study areas.

### Limitation of the study

Despite including participants from three hospitals it might not be applicable for all PLWHA in Ethiopia. This study included only patients on highly active antiretroviral therapy so being on HARRT might underestimate the prevalence of neurocognitive disorder. Since HARRT reduces the prevalence of HAND [72–75].

## Conclusion

There is a high screening prevalence of HIV-associated neurocognitive disorder than those earlier reports in Ethiopia and Africa. The associated factors also vary from that of earlier studies. Therefore, this study indicates the need for formulating preventive cognitive health programs and policies for patients with HIV/AIDS.

### Ethics approval and consent to participate

Ethical clearance was obtained from an Institutional Review Board (IRB) of College of Medicine and Health Sciences, Arba Minch University & permission letter was obtained from Gamo Gofa Zonal health department and respective hospitals.

The nature of the study was fully explained to the study participants to obtain oral consent in the study and any information was kept confidential. The purpose of the study and its consequences were fully explained to the participants like the service they will get from the Hospital will be the same whether they participate in the study or not. In addition, they can stop and go if they don’t feel comfortable even in the middle of the interview. And this statement is approved by the ethics committees/IRBs. In addition, this research didn’t take any sample from the patient (like blood, urine, stool or other invasive procedures). And verbal consent was taken from the study participants in this study. Any personal identifiers were not included in the questionnaire. Any participant, who was screened as having HIV-Associated neurocognitive disorder, was recommended to be evaluated further for possible treatment and follow up evaluations back to the clinicians.

## Supporting information

S1 File Orginal spss. (SAV)

## Abbreviations

AOR: Adjusted Odds Ratio
AIDS: Acquired Immune Deficiency Viruses
ART: Anti-retroviral therapy
BMI: Body Mass Index
cART: combined antiretroviral therapy CDC, Center for Disease Prevention and Control
CI: Confidence
COR: crude odd ratio
HAART: Highly Active Antiretroviral Therapy
HAND: HIV-Associated neurocognitive disorder
HIV: Human Immuno deficiency virus
IHDS: International HIV Dementia Scale
IRB: Institutional review board
PLWHA: People Living With HIV/AIDS
SSA: Sub Saharan Africa
WHO: World Health Organization
